# Relationship between migraine and malocclusion

**DOI:** 10.1186/1129-2377-14-S1-P136

**Published:** 2013-02-21

**Authors:** M Takeuchi, M Kato, J Saruta, K Tsukinoki, H Igarashi

**Affiliations:** 1 Department of Craniofacial Growth and Development Dentistry, Kanagawa Dental College, Japan; 2 Department of Oral Pathology, Division of Salivary Gland Health Medicine, Kanagawa Dental College, Japan; 3 Department of Internal Medicine, Yokohama Medical and Dental clinic, Kanagawa Dental College, Japan

## Introduction

Temporomandibular joint (TMJ) is acting as the only diarthrodial joint in the crania. Therefore, disorders of muscles and TMJ are frequently caused by disharmony of occlusion. It generally showed patients malocclusion and temporomandibular disorders (TMD) treatment of a large number of patients resulted in significant improvement in the physiological state of the general condition and some indefinite complaints including the primary headache.

## Objective

It has been presumed that there is an intimate relationship between TMD and migraine. There is an occlusion which may seriously affect the condyle position of the background for this relationship. The aim of this study is to characterize the occlusion of patients with migraine.

## Subjects and methods

Consecutive 60 female patients with migraine aged 30s-40s attending a headache clinic were studied. Headache diagnosis was based on IHCD-2. We canvassed by using a questionnaire about their general condition and TMD, took photographs and impressions of their occlusion. Based on these materials, we summarize the characteristics of headache patient's occlusion and TMD. This study was approved by the Ethics in Research of the Kanagawa Dental College.

## Results

60 migraine patients (mean age 40 years) and 40 healthy controls (mean age 30 years) completed a baseline questionnaire and occlusal classificasion from models. The mean showed a statistically significant difference (P value 0.05) in the TMD symptoms and occlusal classificasion between headache patients and healthy controls.

## Conclusions

Headache patients suffer from TMD more frequently and have occlusion type is Angle's class' tendency than healthy controls. Relationship between the primary headache and malocclusion suggest a potential for expansion of headache treatment, need further investigations.
